# Permanent health education in a nursing technician course^*^

**DOI:** 10.1590/1980-220X-REEUSP-2021-0276

**Published:** 2022-04-01

**Authors:** Fernanda Juliano de Lima, Letícia Lopes Dorneles, Marta Cristiane Alves Pereira, José Renato Gatto, Fernanda dos Santos Nogueira de Góes, Rosangela Andrade Aukar de Camargo

**Affiliations:** 1 Universidade de São Paulo, Escola de Enfermagem de Ribeirão Preto, Programa de Pós-Graduação em Saúde Pública, Ribeirão Preto, SP, Brazil.; 2 Universidade de São Paulo, Escola de Enfermagem de Ribeirão Preto, Departamento de Enfermagem Geral e Especializada, Ribeirão Preto, SP, Brazil.; 3 Faculdade São Luís de Jaboticabal, São Paulo, Brazil.; 4 Universidade de São Paulo, Escola de Enfermagem de Ribeirão Preto, Programa de Pós-Graduação em Enfermagem Fundamental, Ribeirão Preto, SP, Brazil.; 5 MacEwan University, Center for Teaching and Learning, Edmonton, Canada.; 6 Universidade de São Paulo, Escola de Enfermagem de Ribeirão Preto, Departamento de Enfermagem Materno-Infantil e Saúde Pública, Ribeirão Preto, SP, Brazil.

**Keywords:** Education, Professional, Education, Continuing, Learning, Education, Nursing, Educación Profesional, Educación Continua, Aprendizaje, Educación en Enfermería, Educação Profissionalizante, Educação Continuada, Aprendizagem, Educação em Enfermagem

## Abstract

**Objective:**

To assess the understandings of a pedagogical intervention on the Brazilian National Policy of Permanent Health Education targeted at secondary technical and vocational nursing students.

**Method:**

Applied, pedagogical intervention study conducted with twenty-three students of a secondary technical nursing course; questionnaires, focal group, and thematic content analysis were employed.

**Results:**

Intervention, collectively built by manager, nursing teachers, and researchers, is assessed to have led to a problematization of the concepts of education and continuing and permanent education. The following thematic categories emerged from the analysis: Prior knowledge of students and understandings of the classroom intervention; Relation between permanent education and educational welcome in health units; Ethics concerns and the articulation of care practice and theory; and Work process and approximations to permanent health education.

**Conclusion:**

The pedagogical intervention is assessed to have favored the critical reflection of the aspiring nursing technicians on permanent health education and the need for a collaborative pedagogical planning for aligning the health team’s work process.

## RESUMO

**Objetivo:**

Avaliar as compreensões de uma intervenção pedagógica sobre a Política Nacional de Educação Permanente em Saúde em estudantes da educação profissional técnica de nível médio em enfermagem.

**Método:**

Pesquisa aplicada, do tipo intervenção pedagógica, realizada com vinte e três estudantes de curso técnico de enfermagem; utilizaram-se questionários, grupo focal e análise de conteúdo temática.

**Resultados:**

Avalia-se que a intervenção, construída coletivamente entre gestor, professores e pesquisadores, oportunizou a problematização dos conceitos de educação e educação continuada e permanente. Da análise emergiram as categorias temáticas Conhecimento prévio dos estudantes e compreensões da intervenção em sala de aula; Relação entre educação permanente e acolhimento pedagógico nas unidades de saúde; As inquietações sobre a ética e a articulação teórico-prática no cuidado; e Processo de trabalho e as aproximações com a educação permanente em saúde.

**Conclusão:**

Avaliou-se que a intervenção pedagógica favoreceu a reflexão crítica pelo futuro técnico de enfermagem sobre a educação permanente e a necessidade do planejamento pedagógico colaborativo para o alinhamento do processo de trabalho da equipe de saúde.

## RESUMEN

**Objetivo:**

Evaluar las comprensiones de una intervención pedagógica sobre la Política Nacional de Educación Permanente en Salud de Brasil en estudiantes de educación profesional técnica de nivel medio en enfermería.

**Método:**

Investigación aplicada, de tipo intervención pedagógica, realizada con veintitrés estudiantes de un curso técnico de enfermería; se utilizaron cuestionarios, grupo focal y análisis de contenido temático.

**Resultados:**

Se evaluó que la intervención, construida colectivamente entre gestores, profesores e investigadores, permitió problematizar los conceptos de educación y educación continua y permanente. Del análisis surgieron las categorías temáticas Conocimiento previo de los estudiantes y comprensión de la intervención en la clase; Relación entre la educación permanente y la acogida pedagógica en las unidades de salud; Preocupaciones por la ética y la articulación teórico-práctica en la atención; y Proceso de trabajo y las aproximaciones a la educación permanente en salud.

**Conclusión:**

Se evaluó que la intervención pedagógica favoreció la reflexión crítica de los futuros técnicos de enfermería sobre la educación permanente y la necesidad de planificación pedagógica colaborativa para alinear el proceso de trabajo del equipo de salud.

## INTRODUCTION

In Brazil, Permanent Health Education (PHE) is a political and pedagogical choice and must be understood as both a teaching and learning practice and a health education policy. This form of education has become a legal framework in the Unified Health System (*Sistema Único de Saúde* – SUS) for training and developing healthcare workers through National Health Council Resolution n. 353/2003 and MS/GM Ordinance n. 198/2004, whose premises are: 1) the articulation between teaching, work, and citizenship; 2) relationship between education, sector management, healthcare, and social participation; 3) construction of the SUS network as a space for professional education; 4) the recognition of loco-regional bases as territorial political units, in which education and service structures must be in ‘co-operation’ to formulate educational strategies for qualifying the organization of care into care lines, strengthening social control, and investing in intersectorality. Subsequently, GM Ordinance n. 1.996/2007 established the devices for implementing the National Policy of Permanent Health Education (*Política Nacional de Educação Permanente em Saúde* – PNEPS), adjusting it to operational directives and to the *Pacto pela Saúde* regulation. These Ordinances focus on qualified worker training by valuing multidisciplinary professional practices and the social aspect of educational actions potentialized by collective work, in addition to the necessary specific technical training^(1–2)^. Conceiving PHE as a value is outlining the curricular structure of courses from the perspective of integration between teaching, service, and community, aiming at a transformation of the health model for the consolidation of SUS by articulating themes such as integral care, humanization, and teamwork/interprofessionality. The concept of PHE is aligned to the premises of integral training envisioned by a democratic project of society and education, focused on criticality, autonomy, and citizen responsibility^(1,3)^. However, as in most health undergraduate courses, studies point out that Secondary Technical and Vocational Nursing Education (STVNE) has been hegemonically facing the requirements of instrumental work training for the neoliberal economy. Frequently, STVNE is modulated by utilitarian deals with the health units, guided by a technically oriented education project and by the absence of reflection on the individual/social reality and action^(3–4)^.

To verify the relationship between PNEPS and STVNE in the literature, a search was conducted on the Virtual Health Library with descriptors “*Educação Permanente* AND *Educação Profissionalizante* AND *Enfermagem*” (i.e., the Portuguese terms for Permanent Education, Vocational Education, and Nursing, respectively). Seventeen articles and four theses were identified and, after the inclusion criteria were applied (available complete articles produced in Brazil in the last ten years), no study was observed to discuss this relation.

This type of training requires paradigmatic innovations; thus, this study proposes an approximation of STVNE with the PNEPS, antagonistic to the technically oriented training model in this teaching level. This innovation may (re)signify relations and practices that contribute to a higher quality of health for the population, which is to be covered in Nursing Technician (NT) training. This study also covers aspects of the production of subjectivity and technical and thinking skills from the perspective of training in and for the Brazilian Unified Health System (SUS)^(5)^, which are still distant from STVNE^(3–5)^. This study envisions the collective construction of knowledge of PHE by problematizing care practices in their routine, with the inclusion of STVNE students^(5)^. This must translate into a collaborative pedagogical planning to achieve the strategic goals of SUS^(1)^. Students are emphasized to be actors who interact for long hours with health teams and are frequently perceived as support in work schedules^(4)^. Given this need, which is pointed out both empirically and by the area’s scientific literature, a Pedagogical Intervention (PI) on PNEPS for STVNE students was planned, aiming at providing approximations to this theme and answering the following research question: What are the students’ understandings of this PI? This study aimed thus at assessing the understandings of a pedagogical intervention about the Brazilian National Permanent Health Education Policy by secondary technical and vocational nursing students.

## METHOD

### Design of Study

Applied, qualitative, pedagogical intervention study based on the Consolidated Criteria for Reporting Qualitative Research (COREQ) involving planning and implementation of an interference aimed at advancing the learning processes of aspiring Nursing Technicians (NT) on the PNEPS and subsequent assessment of their understandings of this interference^(6)^.

### Local

The study was conducted in a secondary public Nursing school which offers technical nursing courses in the State of São Paulo, Brazil, linked to a public university hospital. Concerning alumni profile, the school is committed to training professionals who face a reality of constant changes and contradictions, creating a meaningful learning opportunity for the development of thinking skills which enable working knowledgeably to succeed in this adverse reality. To establish a relationship and to plan the PI with the direction and the vocational school’s educators, the research site was approached in four meetings, when reflections on the theme of PNEPS were made to enable teachers to understand its necessity in the training process of these professionals, since this policy is not included the school’s Political and Pedagogical Project (PPP). The discussions attempted at answering the following questions: What would the students be taught? Who would develop the activity? When would be the best moment to teach this class? Where would it be taught? Why is this theme relevant for NT training? Finally, how would it be taught? To deepen this discussion, the animated infographic “Permanent Health Education” – https://www.youtube.com/watch?v=2-E2We4CjdU – was shown; it was produced by the project’s supervisor in a different study dealing with the concepts of Continuing Education (CE) and Permanent Health Education (PHE). Finally, the classroom intervention on PNEPS was decided to be developed by the main researcher and supervisor of the Collective Health course with the STVNE’s educators; after the theoretical internships were concluded, including Women and Child Health in the health units, the formation of a focal group (FG) was agreed upon with the study participants and researchers. Related themes which were previously studied in the 45 hours of the Collective Health course are emphasized to include: the constitution of SUS; natural history of disease; several infectious and parasitic diseases; health surveillance; and National Immunization Policy. Teaching strategies were identified to include, in addition to lectures, seminars and case studies. In the PPP, the school manifests the necessity of trying to move from a culture of transmission of information into a culture of interaction with reality.

### Data Collection

The convenience sample for this study comprised STVNE students who had taken the Collective Health course and who subsequently took theoretical internships including Women and Children Health, both in primary and hospital care. The students were contacted in person and in groups by the researchers for a presentation of the study rationale. This course was chosen due to concentrating contents on SUS. Out of twenty-five students, none declined participation; however, two were excluded for being absent from the class on PNEPS. The twenty-three students agreed to participate and signed the Informed Consent Form (ICF).

### Data Collection and Treatment

The data collection was performed in two different moments between March and June 2020 by the main researcher, a nurse who holds an MSc in Health Sciences and has qualitative research training, and by the project supervisor, a permanent and vocational education researcher who is experienced in qualitative approach. The study included three questionnaires and a Focal Group (FG). The questionnaires were applied during the class, with the presence of the nursing educator responsible for the course, from a position of observing participant. The first questionnaire demanded the participants’ sociodemographic data; the second, their prior knowledge of the concept of education, PE, and PHE; the third, the cognitive knowledge acquired from the PI. All the questionnaires had been analyzed by specialists. The information was organized into three different Excel spreadsheets. Subsequently, a FG was performed with subgroups comprising 11 and 12 participants, respectively, supported by the methodological premises of Minayo and Costa^(7)^. The qualitative aspect of the technique lies on interaction and opinion sharing among participants when an individual reflection may move other participants, enabling mapping convergence and divergence on the theme^(7)^. The FG had a mean duration of 1 h 45, was recorded in audio, and, subsequently, transcribed and validated by the participants. The field notes systematized the classroom experiences, the observations, and the reflections. The FG had the objective of identifying the understandings of the intervention of PHE and had the following guiding question: When taking internships in the several primary and hospital healthcare institutions after the class on PHE, which experiences related to this theme were meaningful to you and the group? The class and the FG were conducted by the main researcher and by the supervisor, whose experience is validated for this type of approach; they mediated discussions, reflections, and the synthesis of knowledge. Concomitantly, a literature review was performed for scientific depth on this theme; the review supported the PI. Searches with Health Science Descriptors were conducted by crossing the terms “*Educação em Enfermagem*” (Nursing Education), “*Educação Permanente*” (Permanent Education), “*Educação Profissionalizante*” (Vocational Education), and “*Pessoal técnico de saúde*” (Technical health staff).

### Pedagogical Intervention Plan

The educational activity was planned with the objective of presenting the PNEPS to students and encouraging reflection on the concepts of PHE and Continuing Education (CE). It was structured through the dialectic methodology^(8)^ and oriented by the understanding that knowledge is built by people in their relation with others and with the world, so that contents are explored in three interdependent pedagogical moments: 1) synchresis or knowledge mobilization, when real-life situations and questions problematizing the theme are presented to the participants for them to express their initial comprehensions/ concepts; 2) analysis or (re)construction of knowledge, when there is a confrontation between prior and new knowledge presented with the objective of favoring dialectical relations; 3) oral and written knowledge syntheses established through discussions among participants and based on the theoretical and practical framework^(8)^. The following activities were scheduled: participant presentation; identification of the prior knowledge of students on PHE through an individual questionnaire; screening of the animated infographic “Permanent Health Education”; discussion in small groups of individual answers and elaboration of a collective answer to the same questionnaire; presentation of the collective answers to the larger group with all students and problematization; final panel with a synthesis of the constructed knowledge; and final assessment of the activity.

### Data Analysis

The information was submitted to Bardin’s content analysis^(9)^, which was organized into three stages: pre-analysis, with a floating reading of the answers to the questionnaires and the transcription of the FG, which was not discussed with the participants; exploration of the material, limited to 126 registration units, leading to 12 codes classified into 4 symbolic categories representing the content; finally, treatment of result, which attempted at apprehending the textual information, and an analysis based on the categories, emphasizing similarities and differences. To guarantee anonymity, the individual statements were identified by codes from A1 to A23 and the groups were identified as GA, GB, GC, and GD. The sociodemographic data were analyzed through descriptive statistics. The partial results of the content analysis were discussed with the participants, a stage which was limited by the participant’s course completion.

### Ethical Aspects

This study was approved by the Research Ethics Committee in Opinion 3.020.365, 2018. All ethical aspects related to research involving human beings were abided by, in conformity with Resolution 466/12 by the National Health Council. The students invited to participate in this study signed the Informed Consent Form after reading it with the researcher.

## RESULTS

The presentation of the results of the PI understandings was synthesized into four subsections. The students participating in this study amounted to twenty-three and were aged 19 to 60; 37.93% of them are men and 62.06% are women; 74% are workers (39% in the health area). In relation to marital status, 44.82% are single and 48.26% are married or in a domestic partnership. Also, 24.13% of them had complete higher education and 24.13% had incomplete higher education. Regarding religion, 34.48% are Catholic, 27.58% are Evangelical, 20.68% have no religion, 13.79% are Spiritist, 3.44% follow Umbanda, and 6.89% did not respond.

### Prior Knowledge of Students and Understandings of the Classroom Intervention

In the classroom, students have shown interest in the PI and were participative, with reflections and experience-sharing which enriched the moment. When recalling the prior individual knowledge of the participants on the concept of education, the idea of collective knowledge construction for citizenship was valued and included discussions about formal education as a social right and the relations with peers in this process of sharing experiences for their social insertion.

*Education is the action of instigating oneself and others about the environment, contributing with experiences, joining others to build practices/knowledge that benefit the whole community* (A5). *Education is a right of all people; individuals acquire knowledge in various levels (primary, secondary, tertiary)* (…) *to be inserted into society and perform their civility, understanding thus their rights and duties for a good social coexistence* (…) *it enables individuals to achieve their personal goals, since the higher the qualification, the better are the job opportunities* (A23).

The concept of CE is perceived as related to professional development beyond initial qualification.

*Continuous education enables professionals to enhance their knowledge, contributing thus to improvements in their activities, with more specific knowledge regarding their profession* (A23).

Whereas the concept of PHE is reportedly unknown by some of the participants, to others it is related to health improvements, with a comprehension of rights and duties, trainings, courses, refreshers aimed at procedures, and other aspects.

*To me, PHE is education that make people aware of their rights and duties regarding their own health in the search for a better quality of life* (A2).

After the presentation of the animated infographic and the discussions, the subgroups modified their responses on previous questions, in a collective construction of the problematized and previously shared knowledge, referring to their immediate understandings of the PI.

*Education is knowledge construction, with principles, e.g.: family, religious, cultural, joining the educational, i.e., educational is learning, understanding, sharing, it is the search for learning and knowledge sharing* (GD). *Continuous education is following a logical sequence of construction and enhancement, expanding current qualification* (GA). *The concept of PHE refers to the identification of problems by multidisciplinary teams, pointing out solutions and activities including courses, talks, and refresher courses for implementation of decisions, aiming at improving healthcare* (GB).

After three months of internship, to raise the a posteriori understandings of the PI regarding PHE, a FG was conducted; in it, students were able to share their significant experiences. This information was used to build three more categories, which better represented the experienced situations and their relationship with knowledge of the PNEPS.

### Relation Between Permanent Education and Educational Welcome in Health Units

The welcoming of students of the health units by the health team was a source of distress and the participants noted that this is related to PHE. There are sectors in which professionals sometimes understand the objectives of the internships and the moment for learning, contributing with an empathetic attitude towards students. The students particularly discussed the attitudes of the medical staff when surprised by their orientations.

*The physicians who worked at the obstetrics center, everything they would do, they would thoroughly explain, as in the case of a hemorrhage, for instance. The nursing technician who worked there also; after the caesarean cut, she would explain the cleaning* (A23).

They also emphasized primary care sites in which the technicians’ attitude was sensitive to the students’ moment of learning and proactively recognized their social role in that situation.

*In the vaccination room, the nursing technicians would be mindful to explain* (…) *the protocols bit by bit* (A14). *Those who were in the pre- and post-consultation* (…) *the nursing technicians were mindful to explain thoroughly how it worked and they were very helpful* (A14).

They also emphasized that the welcoming from health units often frustrated the students’ expectations, because they felt intrusive or alienated by the team while being there to learn.

(…) *what I was learning here was to orient people* (…) *someone will frown at you* (…) *saying “here they come, these crazy people, going up and down and walking into us”* (A22). (…) *the professionals see us as intruders* (…) *they want more employees, not students,* (…) *who give them extra work, and they offer students no opportunities* (A16).

On the other hand, students value the experience of professionals and reflect on how it can contribute to qualified learning. In addition, they emphasize the interest of some people in sharing procedures.

*A nurse who had been working at the hospital for 30 years taught us how to make an enema mixture,* (…) *I tried to absorb this professional’s experience* (…) *enhance my knowledge* (A19).

In addition, an outlook towards integral care was sometimes mentioned when valuing the nursing technician’s actions.

(…) *in the primary unit, there were some people who worked there, who had an integral outlook towards care, like “have you got this little spot checked?”, the nursing technician oriented the patient to have an exam* (A17).

### Ethics Concerns and the Articulation of Care Practice and Theory

In this category, the students pointed out a contradiction between the principles of PHE and their concerns, involving conflicted relations with the health team leading to competition and a non-understanding of the learning process. The team’s approaches towards them were deemed inadequate by the students and the articulation between theory and practice in learning during the work process was considered incoherent. Contradictions between the team’s discourse and practice led to embarrassment and doubt on what is right or wrong. Ethics is questioned due to the disrespect that students feel during the routine of the internship and dubious conducts, such as the ones adopted by the nursing professionals.

*We were in charge of some care procedures and this person – I don’t know if to vex us, I couldn’t tell – would do something we were supposed to do* (A3). (…) *as in the rooming-in, when a father came to me* (…) *he wanted help for his vomiting son. Three nursing technicians were there* (…) *and I told them about it* (…) *whether they could help. And one of the technicians said out loud so that I would hear: “Wow, they think they know everything. You would think they are dying, but it is normal for babies to vomit. It was just a matter of giving attention to the father, who was distressed with the situation”* (A8).

### Work Process and Approximations to Permanent Health Education

The students reflected on how education is necessary for a qualified work process concerning the difficulties of performing procedures with no theoretical knowledge, which could affect safe care.

(…) *I have observed the difficulty of a professional in finding the vein with his glove on. He bit it, removed the tip of the glove, and found the vein through the torn glove* (A23).

Finally, they also reflect on the careers of the units’ professionals, work context, wages, and lack of educational processes.

(…) *there are several factors which lead a professional to certain conducts, such as lack of training* (…) *few benefits* (…) *lack of personnel* (…) *it is positive to ask why it has got to this point* (A3).

The students observed a particular situation during a team meeting to discuss a routine problem, but protagonism was partial, as not everyone participated. The central issue was the lack of adequate equipment to provide healthcare.

*The team gathered to verify the monitors used to obtain the patient’s oxygen saturation* (…) *some of them talked and the others were silent, and only one took hold of the problem and he came across as a bore* (A13).

## DISCUSSION

The immediate understandings of the PI identified an approximation of STVNE students with the PNEPS by resignifying the concepts of education, continuing education, and permanent education through a class mediated by an animated infographic on the theme. Subsequently, other understandings were assessed when the students reflected on the context of the health units in which they were interns and their relationship with PHE. The problems related to pedagogical welcoming and the educational process were emphasized, in addition to the articulation between theory and practice in the work process.

Regarding participant profile, studies corroborate our findings: most of them are working women in varied age groups, married, and often “overqualified”, i.e., with a complete or incomplete undergraduate course^(10–11)^. A recent analysis of wage flexibility and long-term unemployment in six metropolitan areas in Brazil has pointed out that, on average, white individuals, men, and those having a high level of education (over nine years of education) have higher wage rates and face lowest rates of unemployment. In turn, females, non-white people, and those with low education (zero to four years of education) face the opposite situation^(12)^. This study confirms that nursing is an entrance to increased education for women in an attempt to achieve more flexible wage rates and thus a reduced long- term unemployment^(13)^.

Regarding the planning of the PI, which was built from integration between management, educators of the studied school, and the researchers, this study confirmed the need for education to establish collective actions by sharing problems and actions which seek to transform reality. The cooperation and co- responsibility of those involved indicate emancipatory attitudes by valuing and recognizing the possibility of introducing the theme of PNEPS in NT training to widen their interpretation of reality and strengthen formation for SUS^(14–15)^. Historically, the political and pedagogical planning of STVNE has adopted a commercial outlook, setting objectives from a biologistic, medicalizing, procedure-centered approach. Qualification is structured through fragmented courses in which knowledge of basic and clinical areas are dissociated, with a predominance of learning opportunities in the hospital environment^(4,16)^. However, accounting for integral training is presupposing that pedagogical planning is an intentional action which is collectively defined by teachers, management, and student representatives, articulated to a sociopolitical commitment to the actual interests of most of the population. This is expected to educate participatory, responsible, committed, critical, and creative citizens^(14)^. This study shows that this is possible through effort and commitment of all involved with this necessary PI.

The problematization introduced the PNEPS to students when the animated infographic was exhibited, articulating their prior knowledge of the concept of CE and PHE. Continuous education is a personal and professional development of health workers through enhancement of techniques and knowledge related to health practice, whereas PHE is sharing practical experiences through discussions among the different workers in search of critical sense, autonomy, and citizenship^(17–20)^. The responses to individual and group questionnaires have shown that students have consolidated principles of citizenship and social right, but a polysemous outlook to the meaning of PHE. The main ideas of the concepts were the development of work competences, qualification, and training, often in a reduced and acritical manner. On the other hand, the PI has favored the comprehension of these concepts, particularly the necessity of PHE for care qualification, through the collective discussion aimed at deepening the understanding of the work process in search of solutions for their problems^(18)^.

As a whole, the Brazilian educational system predominantly comprised the traditional, renewed, and technicist pedagogical trends, which were hegemonic until the 70’s and, subsequently, those focused on social and political or anti-hegemonic issues were noteworthy, emerging during Brazil’s redemocratization^(19)^. Advancements are perceived with the valuation of the presuppositions of Paulo Freire’s critical pedagogy, which is at the service of social transformations in and for SUS, and particularly PHE, aiming at overcoming social inequalities and searching for social well-being^(19)^. To this end, in this study, students were encouraged to notice the problems they experience, observing, formulating questions, and expressing opinions. This pedagogical strategy has enabled significant learning for the development of intellectual skills of analysis, assessment, and comprehension through exchange of ideas and cooperation among students. This aims at raising awareness about the health workplace and its relations^(18,20)^ by providing students with a critical conscience and a capability of changing their own reality^(21)^.

The a posteriori understandings of the PI were identified particularly through concerns about the educational process experienced during the internships and shared during the FG. Questions about the meaning of pedagogical welcoming in the internships and the meaning of the articulation between theory and practice and their ethical implication in the context of the health team were understood to be an integral part of PHE. This shows the cruciality of a health education encompassing all involved (users, workers, managers, and students) in and for work. This understanding encompasses the necessity of resignifying the training of NT and including them in the process by enabling a reflection on reality which is aware and committed with the enhancement and qualification of SUS^(4,22)^. This training democratically problematizes new forms of care and management, questioning traditional work models which segment care practices^(4,18,21)^. Therefore, a restructuration of the teaching institution, with a PPP contemplating this need through a critical and reflective methodology, and educators who are pedagogically prepared for this objective do not suffice^(16)^. The indispensable condition of pedagogical planning articulated with the health institution from the principles of PNEPS is thus demonstrated. This planning shall have direct implications on the welcoming of students by the health team and on the construction of a collaborative culture committed with education for integral care^(15,20)^.

Studies show that professionals and health units are unprepared to receive and welcome students from the perspective of PHE, either due to structural problems or lack of interest or financial, scientific, or cultural incentive by teaching institutions, complicating this integration^(1,16)^. The results of this study point out that unsystematized pedagogical welcoming, which depends on the individual conscience of each professional of the health team, compromises the ethical and political work process of health units employing integral care. In the absence of daily dialogue promoted by PHE, the health professionals are observed to be unaware of their educational role towards the STVNE students. Some have a clear intention of contributing and cherishing their experiences. The primary units are emphasized to be a valued space and the extent to which NT and nurses in these places try to share knowledge and experiences, such as in the vaccine and pre-consultation room, is noticeable, possibly because approximations with the PNEPS are more frequent. However, students are often left to their own devices or are received with disdain and even sarcasm towards well-founded and safe care, as when a student requested that a child be helped with vomiting, which was neglected, as reported. Surprisingly, they registered a warm reception by medical professionals since these students are frequently invisible in the health context^(16)^.

In professional training, knowledge is notedly built by real situations in the world of work through integration of students in health services, supported by a pedagogical project which must be directed at the reality of the health system, with principles and policies to guide it, targeting humanized integral care^(17)^. Thus, the concept of welcoming, as in SUS, has been recognized as a political and pedagogical directive in the education of young people and adults and implies a dialogic, critical, and sensitive attitude based on bonding, alterity relations, and affection. This concept also involves the recognition of knowledge, experiences, and life stories of students, who attribute meaning to the curriculum, rejecting or reporting all forms of oppression, prejudice, and discrimination that may affect students^(23)^.

From this perspective, this study points out the importance of these teams’ professional conscience of the pedagogical welcoming of aspiring colleagues, which may share their work environment. Solidarity with learners is known to be a foundation of citizenship, whose goal is the common good and whose bridge in health might be PHE. Despite the major responsibility lying on the internship supervisor, collaboration within teams in this inclusive educational process, which acts complementarily, enhances cognitive, procedural, and attitudinal competences of the aspiring NT, as they are involved in the same context and with the same objectives. In addition, team members are role-models, professional references for students, who expect feedback when affiliating to collective actions involving integral care. The absence of spaces for PHE that promote discussion and planning of pedagogical welcoming by the unit’s health professionals has been possibly leading to significant losses in the development of the ethical and political dimension for the formation of NT and their exclusion from purposeful dialogue and, consequently, of the health team in their relations of alterity and affection. Examples include attitudes which humiliate and intimidate students, as found in this study, leading to a lack of courage to question negligent practices^(24)^. The comprehension that NT students must attend to procedure performance is present in many health units, possibly due to a utilitarian understanding of education, mistakenly regarded as workforce directed. In this sense, a study points out that the health team and bedside nurses expect students to fill the professional gap. This view strengthens the dissociation between theory and practice and reduces the internship to the idea of a “practice of theory”, a space for technical instrumentalization, which reinforces the distance between teaching and service^(25)^. On the other hand, this study’s participants report situations of rejection and despise by the nursing team, with attitudes deemed to be disrespectful and to compromise care. This fact is possibly related to the team’s perception that student training is not their job and may often overload their routine^(3,5,16,20)^. The absence of reflection about these questions may limit the apprehension of new meanings to health actions for individual and collective life, inserted in a health model focused on social needs.

The integration between education and service put forth by the PNEPS is essential for the performance of teaching institutions supervising students in the theoretical and practical context; it must be delimited by planning the insertion of these students from the perspective of integral care^(1,15,21)^. However, one of its major challenges is the lack of institutionalization and an incipient participation of several actors, as shown by this study, which was clearly noted by participants as absent in the units where their internships were conducted, including a reported professional underqualification. They also noted that team meetings are rare and the involvement of workers is partial and stagnant. On the other hand, they mentioned the need to consider the problematization of the context in which the team acts, its challenges, and limitations to extend the established work process and possible social pressures and work overload. This is notedly an exercise in which they correlate what they learned during the PI with their experiences in the context of practice. The concept of PHE implies a critical and reflective educational process concerning the reality of all health actors, including students, with deals favoring interprofessional and multiprofessional integral care through an interdisciplinary logic^(1,21,24)^, a strategy which seeks to establish horizontal relations within the teams^(18)^. The complexity of this proposal depends on a syllabus which is contextualized to services and a PPP which is coherent and cohesive with these principles, in addition to qualified educators with political and pedagogical preparation^(20–22)^. This study identified in the PPP of the studied school approximations with the need for training NT to face a contradictory reality, to be resignified with critical conscience in a dynamic which reflects the routines of PHE. However, this is the start of a construction which requires permanent involvement from all protagonists of health training and care.

## CONCLUSION

The PI was assessed to have provided meaning to the PNEPS, since it enabled a critical reflection of students by correlating the concepts which were problematized in the classroom with the experiences of their internships. Among other aspects, they understood that PHE signifies a pedagogical welcoming by the health team, in situations in which individual initiatives, among the NT of primary care, produced affection and changes, since they included students in real-life care problems and provided learning opportunities. In other situations, they felt rejected and intimidated by the professionals for bothering them when asking questions about users or acting with no sensibility or with inexperience. In these places, they observed the meaning of knowledge devaluation and discomfort due to a lack of bonds, since they started to understand that learning depends on interactions, solidarity, and mutual comprehension expressed from listening and dialogue, which are the foundations of the implementation of PHE. Given these perceptions, a joint pedagogical planning by both health and educational institutions is necessary, with the involvement of health team, educators, school, and health managers, aligning pedagogical actions which contemplate the training principles of the PPP. Finally, the inclusion of PNEPS on the curricular structure for NT training is recommended, with the objective of developing a critical conscience of the educational process in and for ethical and political work of STVNE students. This study is understood to have indicated paths to paradigmatic innovation for citizenship education targeted at SUS, to the detriment of a technicist and impersonal education. It is considered to be limited by providing solely the students’ outlook of the studied object. Studies involving educators and the health team are required.

## ASSOCIATE EDITOR

Vilanice Alves de Araújo Püschel
